# A Review of the Processes, Parameters, and Optimization of Anaerobic Digestion

**DOI:** 10.3390/ijerph15102224

**Published:** 2018-10-11

**Authors:** Jay N. Meegoda, Brian Li, Kush Patel, Lily B. Wang

**Affiliations:** 1 Department of Civil and Environmental Engineering, New Jersey Institute of Technology, Newark, NJ 07102, USA; lskbr02@yahoo.com (B.L.); kap68@njit.edu (K.P.); 2 The Pingry School, Basking Ridge, NJ 07920, USA; lilybwang@gmail.com

**Keywords:** anaerobic digestion, biogas, energy, food waste, optimization, parameters, processes

## Abstract

Anaerobic digestion is a technology that has been used by humans for centuries. Anaerobic digestion is considered to be a useful tool that can generate renewable energy and significant research interest has arisen recently. The underlying theory of anaerobic digestion has been established for decades; however, a great deal of current research is directed towards the optimization of anaerobic digestion under diverse digestion conditions. This review provides a summary of the processes underlying anaerobic digestion, commonly-utilized measurements of anaerobic sludge, operating parameters of anaerobic digesters, and methods of acceleration and optimization used to improve process efficiency. Recent developments in addition to older research are considered to provide a general but comprehensive summary of accumulated knowledge in the theory of anaerobic digestion, as well as considerations in the efficient operation of digesters. We have determined that the numerous factors pertinent to the design and operation of batch-based anaerobic digesters must each be considered to ensure the maximum efficiency and cost-effectiveness of a digester provided its respective operating conditions.

## 1. Introduction

Historical evidence from Assyria and Persia indicates the use of biogas for heating bathing water as early as the 10th century B.C.E. (Before Common Era, i.e., B.C.) [1]. In the Middle Ages, Jean Baptiste van Helmont observed the production of combustible gas from the decomposition of organic matter in lakes [2]. Later on, Alessandro Volta conducted a series of experiments on combustible gas that was collected from marsh sediments, observing a direct correlation between degraded organic matter and gas production [3]. In 1808, Humphry Davy discovered that anaerobically digested cattle manure produced methane, which aroused the possibility of producing combustible gas from manure [1].

**Figure 1:**
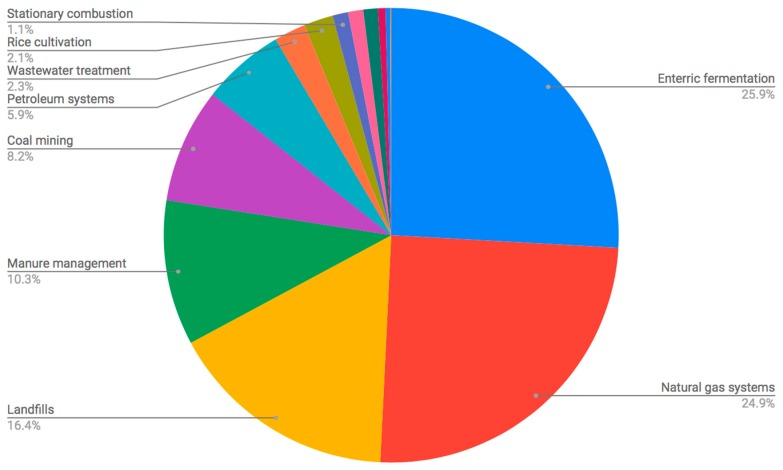
The 2015 methane emissions sources in the United States by percentage [4].

Current waste management practices in the United States allows for significant methane emissions. In 2015, landfills, animal waste treatment, and wastewater treatment constituted the third, fourth, and seventh largest sources of methane emissions, respectively (Figure 1), collectively being culpable for roughly 45% of methane emissions as a carbon dioxide equivalent [4]. However, infrastructure that supports anaerobic digestion is not only able to reduce greenhouse gas emissions, but can also generate renewable energy and divert organic wastes from landfills [5]. Currently, there exist over 2000 facilities producing biogas in the United States; however, that number could climb to over 11,000 provided that proper support is afforded [6]. Nonetheless, anaerobic digestion still faces a series of social and economic obstacles that prevent its full potential from being leveraged, although market conditions are becoming increasingly favorable to anaerobic digestion [7,8].

The objective of this research is to develop a small-scale anaerobic digester for schools and restaurants to compost food waste while recovering biogas to meet their energy needs. The food waste degradation will be optimized via anaerobic digestion followed by aerobic composting after biogas production is reduced. The expected prototype that would result from this research would be a food waste digester containing the following chambers: (1) a food waste shredder, (2) an airtight and heated anaerobic digester with an auger for mixing, (3) an aerobic chamber with an auger, and (4) a compost storage bin. The generated biogas can be connected to the gas line via a pressure equalizer, and excess effluent can be discharged to the sewers.

**Figure 2:**
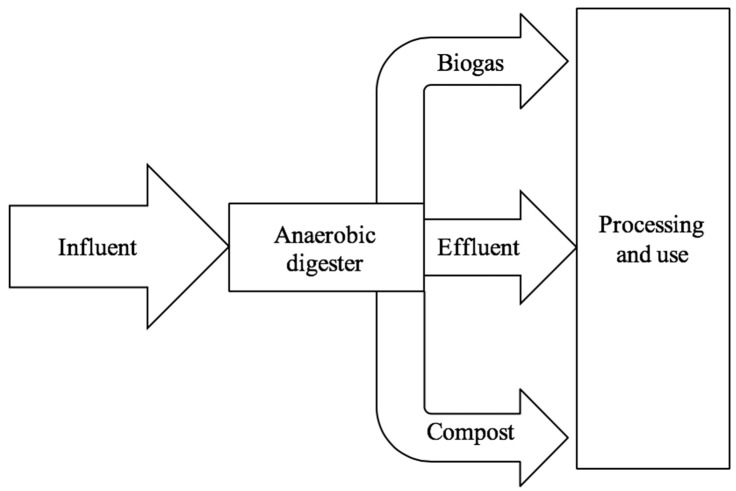
The anaerobic digestion influent and output streams.

This review aims to provide an overview of several selected topics that have been extensively investigated in anaerobic digestion. Conceptual information and research findings aim to provide a comprehensive summary of developments in anaerobic digestion, both early and recent. Section 2 provides an overview of the four stages of anaerobic digestion, summarizing the biochemistry of anaerobic digestion in addition to providing a brief discussion of the inhibition sources. With the stages of anaerobic digestion introduced, Section 3 proceeds to review some of the tests used in quantifying anaerobic digestion, particularly of the sludge and substrates used. Section 4 then summarizes several areas design considerations for digesters and further delves into a discussion of process efficiency. Finally, Section 5 provides an overview of several common pretreatments that can be used to increase the process efficiency and reduce digestion times. Aspects of this accumulated knowledge can be applied to develop small-scale single-stage batch digesters. Figure 2 provides a schematic representation of such a digester.

## 2. Stages of Anaerobic Digestion

**Figure 3:**
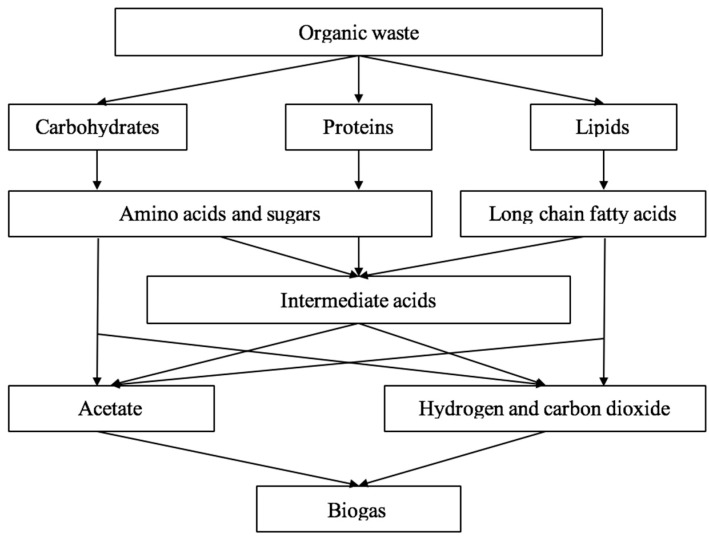
The simplified scheme of pathways in anaerobic digestion [10].

The process of anaerobic digestion takes place through four successive stages: hydrolysis, acidogenesis, acetogenesis, and methanogenesis; the anaerobic digestion process is dependent on the interactions between the diverse microorganisms that are able to carry out the four aforementioned stages [9]. In single-stage batch reactors, all wastes are loaded simultaneously, and all four processes are allowed to occur in the same reactor sequentially; the compost is then emptied after at the conclusion of a given retention period or the cessation of biogas production [9]. Figure 3 depicts a simplified flow of the four digestion stages described below.

### 2.1. Hydrolysis

Anaerobic digesters typically encounter organic biomass that contains complex polymers which are inaccessible to microorganisms without being further broken down through hydrolysis or pretreatments [10]. As a result, the process of hydrolysis serves the purpose of rendering organic macromolecules into its smaller components, which in turn can be utilized by acidogenic bacteria.

While hydrolysis can exist as an electrochemical process, in anaerobic digestion, it mostly exists as a biological one. In the process of hydrolysis, hydrolytic bacteria are able to secrete extracellular enzymes that can convert carbohydrates, lipids, and proteins into sugars, long chain fatty acids (LCFAs), and amino acids, respectively [11]. After enzymatic cleavage, the products of hydrolysis are able to diffuse through the cell membranes of acidogenic microorganisms [12]. However, it is important to note that certain substrates, such as lignin, cellulose, and hemicellulose, may find it difficult to degrade, and can be inaccessible to microbes due to their complex structures; enzymes are often added to enhance the hydrolysis of these carbohydrates [13].

Hydrolysis can be a rate-determining step, although prior research has also demonstrated that methanogenesis might exist as a rate-determining step depending on the ratio of hydrolytic to methanogenic microorganisms [14,15].

Due to the importance of hydrolysis in the kinetics of anaerobic digestion, a great deal of attention has been turned towards methods for expediting hydrolysis in anaerobic digesters. A variety of waste pretreatment options are being researched and utilized to optimize hydrolysis, especially for digesters that digest heavily lignocellulosic wastes [16].

Generally speaking, hydrolysis has, on its own, an optimum temperature between 30–50 °C and with an optimum pH of 5–7, although there is no evidence of improved hydrolytic activity below a pH of 7 [17].

### 2.2. Acidogenesis

By absorbing the products of hydrolysis through their cell membranes, acidogenic microorganisms are able to produce intermediate volatile fatty acids (VFAs) and other products. VFAs constitute a class of organic acids such as acetates, and larger organic acids such as propionate and butyrate, typically in a ratio varying from 75:15:10 to 40:40:20 [18]. Even then, smaller amounts of ethanol and lactate may be present [12]. The specific concentrations of intermediates produced in the acidogenesis stage may depend on the conditions of the digester; it has been reported that VFA concentrations can fluctuate significantly for digesters operating at different pH, with different studies presenting seemingly contradictory results [19,20].

As opposed to other stages, acidogenesis is generally believed to proceed at a faster rate than all other stages of anaerobic digestion, with acidogenic bacteria having a regeneration time of fewer than 36 h [21]. With the rapidity of this stage in mind, it is important to note that while the production of VFAs creates direct precursors for the final stage of methanogenesis, VFA acidification is widely reported to be a cause for digester failure [22]. A somewhat similar anaerobic process is present in bokashi composting, a composting practice in which food wastes and a microbial inoculant are left to degrade anaerobically, creating a highly acidic final product that can be used as a liquid and dry fertilizer [23].

Finally, in protein-rich wastes such as sewage wastewaters, it fits to examine the process of VFA production from amino acids. Amino acids generally degrade into VFAs in pairs via the Stickland reaction, with single amino acid degradation also possible when hydrogenotrophic bacteria are present, although this latter process is known to be slower than the Stickland reaction [24]. One important product of the amino acid breakdown is the production of ammonia from deamination, which, at sufficiently high concentrations, is known to also be an inhibitor of anaerobic digestion [24,25].

### 2.3. Acetogenesis

With the production of acetate through acidogenesis, a portion of the original substrate has already been rendered into a substrate suitable for acetoclastic methanogenesis [26]. However, other produced higher VFAs have yet to be made accessible to methanogenic microorganisms. Acetogenesis is the process by which these higher VFAs and other intermediates are converted into acetate, with hydrogen also being produced [27].

The hydrogen that is produced during acetogenesis broaches the discussion of an interesting syntrophic relationship that is present in the anaerobic digestion—hydrogen interspecies transfer. While acetogenesis is a producer of hydrogen, an excessive partial pressure proves to be deleterious to acetogenic microorganisms [28]. However, due to the presence of hydrogenotrophic methanogens, hydrogen is able to be rapidly consumed while maintaining hydrogen partial pressures at a level favorable to acetogenesis by creating an exergonic reaction [29].

At the same time, lipids undergo a separate pathway of acetogenesis via acidogenesis and β-oxidation, where acidogenesis produces acetate from glycerol and β-oxidation produces acetate from LCFAs [30]. With this in mind, it would be useful to be mindful that only LCFAs with an even amount of carbons can degrade to acetate; LCFAs with an odd amount of carbons are first degraded to propionate [30].

### 2.4. Methanogenesis

Methanogenesis marks the final stage of anaerobic digestion, where accessible intermediates are consumed by methanogenic microorganisms to produce methane [31]. Methanogenic microorganisms represent a group of obligate anaerobic archaea; as a testament to the acute sensitivity of methanogenic microorganisms to oxygen, it was found that 99% of *Methanococcus voltae* and *Methanococcus vannielli* cells had been killed within ten hours upon exposure to oxygen [32].

In addition to a sensitivity to oxygen, methanogenic microorganisms are confined to a small selection of substrates. Typically, acetoclastic methanogenesis from acetate accounts for approximately ⅔ of the methane production, with hydrogenotrophic methanogenesis accounting for approximately the remaining ⅓ of the methane production; however, methanogenesis from methanol, methylamines, and formate has also been observed [33,34].

With regards to the environmental needs of methanogenesis, methanogenic microorganisms tend to require a higher pH than previous stages of anaerobic digestion, in addition to a lower redox potential, the latter requisite having caused significant trouble for laboratory cultivation [35]. At the same time, methanogens appear to have a significantly slower regeneration time than other microorganisms in anaerobic digestion, upwards of 5–16 days [21]. However, it has been reported that some hydrogenotrophic species, such as *Methanococcus maripaludis*, have a doubling time of only two hours [36].

While methanogenic species likely constitute the most sensitive of microbial groups present in anaerobic digestion, recent research has suggested that *Methanosarcina* spp. tend to be relatively robust, capable of withstanding ammonia, sodium, and acetate concentrations in addition to pH shocks at levels that would otherwise be detrimental to other methanogenic microorganisms [37].

In batch reactors, the end of methanogenesis is determined when biogas production stops, which can take about 40 days [9]. Evaluations of a sludge’s extent of digestion can be taken from its volatile solids content and its ability to dewater [38].

## 3. Quantitative Evaluations of the Anaerobic Digestion Process

The following subsections describe the commonly-used metrics used in quantitative evaluations of the anaerobic digestion process.

### 3.1. Biochemical Oxygen Demand

Biochemical oxygen demand (BOD) provides a measure of biodegradable organics present in a sludge, and, in turn, can be used as a metric for the overall effectiveness of an anaerobic digester [39]. BOD reflects values from the microbial metabolism of dissolved oxygen in a given sample of sludge over the course of five days. Ultimately, BOD is a value that can be used to determine the amount of dissolved oxygen needed to sustain aerobic microorganisms to in a sludge sample over an experimental period of five days, which in turn can be used to quantify the concentration of biodegradable organics present in sludge [39].

BOD testing is conducted in sealed bottles at a prescribed temperature and in a dark room to prevent any dissolved oxygen production from photosynthesis [39]. Thus, BOD can be obtained from the difference of dissolved oxygen and the start and end of incubation after dilution is accounted for. Alternatively, a variation of BOD, carbonaceous BOD (cBOD), is determined from a similar protocol, except a nitrification inhibitor is added to prevent the oxidation of ammonia, nitrogen, and nitrite [40]. It could be surmised that for sludges with a high protein content, such as sewage wastewaters, cBOD would be able to provide a more accurate measurement of the organics present. A similar measure that used for aerobic digestion of sewage wastes is the oxygen uptake rate, in which a measure of biological activity is obtained from the consumption of oxygen in an aerobic sludge over a given experimental period [41].

### 3.2. Chemical Oxygen Demand

Like BOD, chemical oxygen demand (COD) provides a measure of the oxygen present in a sample of sludge that can be consumed in a reaction with oxidizing agents [39]. In anaerobic digestion, COD typically reflects the number of organics present in a sludge. The efficiency of anaerobic digestion can also be evaluated using COD; COD reduction can be reflective of the amount of degradation taking place within an anaerobic digester, as it reflects the consumption of organics [12].

In COD tests, a sludge is refluxed in excess with a solution of potassium dichromate and sulfuric acid. The use of potassium dichromate obviates the need for a consideration of nitrification, for it is unable to oxidize ammonia into nitrate [42]. Upon completion of a reflux, the quantity of excess potassium dichromate can be determined by a titration against ferrous ammonium sulfate; the final COD value can be determined from the amount of potassium dichromate consumed in the initial reflux [39].

#### Relating Measures of Biochemical and Chemical Oxygen Demand

As described above, COD is typically calculated from a dichromate reflux, a process which can be completed within a few hours. In contrast, testing for BOD, a related but separate measure, typically takes five days, as instead of using strong oxidizing agents to oxidize a sludge sample, BOD testing relies on the use of aerobic bacteria to oxidize the biodegradable organics in a sludge sample. Generally, BOD testing is avoided due to logistical difficulties, especially because of the time needed to complete the test, for obtaining results after five days no longer provides an accurate insight to the present conditions of the digester and is therefore unreliable in making judgments with regards to operational adjustments [42].

As COD measures all organics in a sludge, its value is understandably higher than that of BOD. Thus, the ratio of BOD to COD can be used to represent the biodegradable fraction of a sludge [43].

### 3.3. Carbon/Nitrogen Ratio

The carbon/nitrogen ratio (C/N ratio) of a substrate is a commonly-used characterization of nutrients. Considering the composition of carbohydrates, lipids, and proteins, it stands to reason that the most abundant source of nitrogen in an anaerobic digester would be from the degradation of proteins. Just as carbon is necessary at a certain concentration to provide a suitable substrate for digestion, nitrogen at a certain concentration is also necessary lest the protein formation for microorganisms be compromised [44]. In a study conducted on dairy manure, it was found that increasing C/N ratios led to decreasing methane concentrations in biogas, with an optimum at a C/N ratio of 25:1 [45].

The C/N ratio has also been a subject of attention as co-digestion of multiple substrates has become increasingly utilized. For example, poultry manures have been known to have a relatively low C/N ratio due to a high ammonia content, possibly due to urea; as such, carbon-rich substrates such as straw may be co-digested to obviate the possibility of ammonia inhibition [46,47]. In a more recent study conducted with mesophilic and thermophilic digesters for the co-digestion of dairy manure, chicken manure, and rice straw, an optimal methane potential and reduced ammonia inhibition were observed at C/N ratios of 25:1 for mesophilic digesters and 35:1 for thermophilic digesters [48].

### 3.4. Theoretical Methane Yield

In summarizing the four stages of anaerobic digestion, there have been attempts to quantify the theoretical methane yield for a given substrate. Working under the assumption that all substrate is converted to either carbon dioxide or methane, and that the carbon, hydrogen, and oxygen composition of the substrate are known, one could use the following equation and the general gas equation to find a theoretical molar and volumetric output of methane [49]:

$$C_{n}H_{a}O_{b}+\left(n-\frac{a}{4}-\frac{b}{2}\right)H_{2}O\to \left(\frac{n}{2}-\frac{a}{8}+\frac{b}{4}\right)CO_{2}+\left(\frac{n}{2}+\frac{a}{8}-\frac{b}{4}\right)CH_{4}$$

However, this model assumes that only methane and carbon dioxide are produced, in addition to discounting the effects of inhibition. However, it is known that neither of these assumptions is possible under actual operating conditions. To account for additional products, a variation of the above equation was proposed, which accounted for the presence of nitrogen and sulfur in wastes and the production of ammonia and hydrogen sulfide [50]:

$$\;C_{n}H_{a}O_{b}N_{x}S_{y}+\left(n-\frac{a}{4}-\frac{b}{2}+\frac{3x}{4}+\frac{y}{2}\right)H_{2}O\;\;\to \left(n-\frac{a}{4}+\frac{b}{2}+\frac{3x}{4}+\frac{y}{2}\right)CO_{2}+\left(n+\frac{a}{4}-\frac{b}{2}-\frac{3x}{4}-\frac{y}{2}\right)CH_{4}+xNH_{3}+yH_{2}S$$

While these measurements may not be useful in calculating methane yields in a continuous digester, they may be useful for making comparisons between experimental and theoretical methane yields in bottle assays. Deviations from the theoretical methane yield are likely; the dynamic nature of anaerobic digestion can easily cause digester upsets and process failures. However, the rate and quantity of biogas generation may depend on the amount of organics left, the availability of the organics for digestion, and the over-accumulation of inhibitory compounds, and sudden changes in the digester pH [51].

### 3.5. Volatile Solids

Volatile solids (VS) is generally treated as a measurement of the organic fraction of total solids, although a more accurate description would be the amount of a matter in a sludge that is lost on ignition [52]. The VS content is determined by the igniting the remaining solids produced from total solids measurement at 550 °C, though some volatilization may have already occurred during the measurement of the total solids [53].

Like COD, VS can be treated as a measurement of organics in water, although the former is a more accurate measure. Nonetheless, both measurements can be used as a basis for determining the organic loading rate of a digester, which is further discussed [52]. Like BOD/COD, VS reduction is also treated as a measurement of digester efficiency by itself and can also appear as a component of other measurements [54].

## 4. Digester Design Considerations

### 4.1. Hydraulic Retention Time

Hydraulic retention time (HRT) refers to the mean length of time that liquids remain in a digester. HRT, which often appears as θ in literature, can be calculated as the quotient of digester volume, V, and flow rate of a digester, Q [55]:

$$\theta =\frac{V}{Q}$$

As a measure that is related to the loading rate, a shorter HRT corresponds to a higher loading rate. As such, shorter HRTs are known to be associated with VFA acidification, which could bring inhibitory effects [56]. Nonetheless, shorter HRTs allow for increased process efficiency and decreased capital costs, although longer HRTs are necessary for the digestion of lignocellulosic wastes [57]. Generally, mesophilic digestion can be accomplished within 15–30 days [58]. However, after cost-benefit analyses of municipal wastes, the highest benefit is found for digesters operating on a low loading rate and high HRT [59].

### 4.2. Loading Rate

The loading rate of a digester refers to the amount of organics fed to a digester per day in continuous digesters. Overloading a digester may cause upsets in that waste is quickly hydrolyzed and acidified, thus creating an over-accumulation of VFAs, which has the potential of inhibiting methanogenesis, thus disrupting the anaerobic digestion process [60]. For example, it has also been demonstrated that olive mill waste digesters operating on an increased loading rate saw a decline in decreasing pH, COD reduction, and most importantly, the rates of biogas production [61]. In a high solids batch digesters, the loading rate can be only half of other single-stage reactors, which in turn necessitates a greater land footprint [9].

Studies conducted on overloaded grease waste digesters found that rapid shocks in the loading rate were able to cause shifts in microbial populations, with methane yields returning to normal levels after developing a tolerance to higher loading rates [62]. It has been hypothesized that improved digester performance and resistance to overloading after an initial instance of overloading is due to an increased diversification of methanogenic microorganisms [63].

### 4.3. Total Solids

Total solids (TS) is a measurement of dry matter in a sludge, irrespective of its organic or inorganic nature; it is often articulated in the literature as either a percentage or a concentration [52]. The TS content is determined by the drying of a sludge sample at 103–105 °C in succession until no further change in weight is observed [53].

Along with being an evaluation of influent, TS is an important attribute of digester operation. High-TS anaerobic digestion has received a considerable amount of recent attention, on account of its need for smaller digester sizes and lower heating needs [64]. Furthermore, improved biogas yields were reported in continuous high-TS digesters compared to low-TS digesters operating on the same retention time [65].

### 4.4. Temperature

There exist two main temperature regimes for anaerobic digestion: mesophilic (35 °C) and thermophilic (55 °C). Because mesophilic digestion operates in a lower temperature, digestion at this temperature regime is slower and yields less biogas; however, mesophilic digesters remain attractive because of their lower heater energy costs compared to thermophilic digesters [66].

Thermophilic digestion, on the other hand, operates at a higher temperature. Consequently, reaction rates are increased, leading to a possibility of higher loading rates, in addition to increased biogas production [59,67]. In addition, thermophilic digestion is known to have higher levels of pathogen destruction, which can prove useful in certain jurisdictions with regulations on pathogen activity in effluents [68]. At a digester temperature of 53 °C, a 90% decimation time of less than one hour was reported for several pathogens, while a 90% decimation time of several days was reported for the same pathogens in a digester operating at 35 °C [69].

Some digesters are dependent on the ambient air temperature, requiring no heating; these digesters often see seasonal fluctuations in methane production [58]. At the same time, research in unconventional temperature regimes has also been conducted, such as the use of Alaskan lake sediments in psychrophilic food waste digestion at −15 °C to +15 °C [70].

## 5. Pretreatments

### 5.1. Biological Pretreatments

Biological pretreatments may include aerobic and anaerobic pretreatments, although these treatments are generally not applied to municipal wastes [71]. White rot fungi have been investigated as a potential biological agent in the pretreatment of lignocellulosic wastes through enzymatic secretions, although the use of fungi runs the risk of long pretreatment times and there exists a proclivity for certain white rot fungi to destroy cellulose [72]. The former disadvantage can be especially troubling, as pretreatment times of several weeks to several months may be necessary for considerable lignin destruction [72].

In addition, temperature-phased anaerobic digestion (TPAD) is another means of biological pretreatment, in which a waste is first digested at thermophilic or hyperthermophilic conditions to promote hydrolysis [73]. TPAD, particularly thermophilic-mesophilic TPAD, is particularly useful in increasing hydrolysis, which in turn translates to greater VS removal and increased methane production [74].

Enzymes produced by industrial fermentation processes can also be used as an accelerant for the pretreatment of lignocellulosic wastes [75]. In food waste acidification reactors, it was found that a mixture of carbohydrates, protease, and lipase in a 1:2:1 ratio was most conducive to VS reduction [76]. However, the efficiency of and long retention times of biological pretreatment has suggested that it is less advantageous than other means of pretreatment [77]. Furthermore, the marginal biogas increases may not be able to justify the expensive cost of enzymes [78].

### 5.2. Chemical Pretreatments

Acidic pretreatment is one form of chemical pretreatment, in which lignocellulosic substrates are broken down into their respective monosaccharides [71]. Furthermore, the acidity associated with this kind of pretreatment can be adjusted to by hydrolytic microorganisms [71]. While acidic pretreatment is able to assist with the degradation of substrates in addition to reducing the time required for digestion, its costs render it less financially effective than utilizing alkaline pretreatments [79].

Alkaline pretreatment, another form of chemical pretreatment, typically entails ammonia or hydroxide compounds [58]. These treatments use reagents which are less caustic than acidic pretreatments and can be conducted at ambient temperature [80]. The principle of alkaline pretreatment is to cause fibers to swell, thus disrupting the structure of lignin and exposing the substrate to enzymatic degradation [63]. As anaerobic digestion occasionally necessitates the addition of alkaline reagents to balance the pH, alkaline pretreatment is a preferred method to acidic pretreatments [81].

### 5.3. Mechanical Pretreatments

The principal objective of mechanical pretreatment is the reduction of the particle size in wastes, thereby increasing the surface area of particles [16]. Ultrasound pretreatment is one method by which this form of pretreatment is conducted, in which the produced cavitation is able to cause cell lysis [82]. At 20 kHz, 80 min of sonification led to the highest biogas production, with longer periods of sonification being observed to be more conducive to VS reduction [83].

Liquid shearing has garnered some degree of research attention, particularly collision plate pretreatment; this method entails jetting a sludge against a smash plate at high pressure, thus causing cell lysis [84]. This pretreatment was able to reduce the HRT of waste activated sludge from 13 days to six days without any hindrance of process efficiency [84]. Nonetheless, it should be noted that research in collision plate pretreatment has been confined to the laboratory scale [73].

Milling is also a mechanical pretreatment by which substrate size can be reduced. Although the fine milling of lignocellulosic waste can translate into better conversion than coarse milling, caution must be taken, for excessively fine particles may run the risk of acidification due to high waste solubility [85].

### 5.4. Thermal Pretreatments

Thermal pretreatment involves exposing wastes to high temperatures a pressure to induce hydrolysis while preventing evaporation [71]. Increased loading rates can be applied to digesters that incorporate thermal pretreatments [86]. Furthermore, cell disintegration and hydrolysis are able to create a sludge that is more biodegradable and allows for more stable digestion [87,88].

The temperature of thermal pretreatment has a bearing on the efficacy of the pretreatment process. Additionally, an excessive temperature could result in the destruction of VS, depleting the available substrate for anaerobic digestion [71]. Furthermore, the increased solubility of carbohydrates and proteins at high temperatures may cause toxic melanoidins to accumulate from the Maillard reaction [89]. However, it has been proposed that the mechanism by which low-temperature thermal pretreatment occurs is via enzymatic hydrolysis [90]. Despite its lower temperature, thermal pretreatment at 70 °C was still able to produce marked pathogen reduction [91].

## 6. Discussion

The current degree of understanding of anaerobic digestion is the fruit of many years of accumulated research. In recent years, a growing interest in renewable and sustainable energy resources has contributed to an unprecedented level of interest in anaerobic digestion research. This review attempts to summarize some of the knowledge produced during this continuing research process, in addition to providing a general overview of some of the techniques and measures used in the quantification of anaerobic digestion.

The biochemistry of anaerobic digestion is one of many fields that contributes to our current knowledge of anaerobic digestion. Anaerobic digestion can be best described as a four-stage process, as wastes are continuously broken down by anaerobic microorganisms and converted into biogas, a mixture of methane, carbon dioxide, and trace gases [92]. Each stage of anaerobic digestion is carried out by different groups of microorganisms, each with their own respective environmental optimum; methanogenesis proves to be a particularly sensitive process, as it is carried out by a small class of strictly anaerobic archaea [21]. Considering the sensitivity of anaerobic digestion, the imbalances caused by the over-accumulation of certain intermediates in a digester can easily cause an inhibition or process failures [93]. As such, the design of single-vessel digester, continuous or otherwise, must be able to take into these factors in order to ensure efficient and thorough digestion, and there should exist methods by which potential upsets in digesters can be detected and rectified. A protocol was developed by our research group to extract samples via needle and evacuated tube throughout the digestion process to detect potential disruptions.

Anaerobic digestion can be quantified by a variety of tests. For example, a substrate used for anaerobic digestion can also be characterized in terms of its C/N ratio, which can provide insight into its suitability for digestion and the likelihood of VFA acidification or ammonia inhibition; as such, a digester design might require the consideration of co-digestion as a means by which the C/N ratio is balanced [92]. At the same time, other evaluations of sludge, such as VS, BOD, and COD can be useful metrics in both the lab-scale and at-scale testing of anaerobic digesters. Factors that affect the efficiency of anaerobic digestion must also be considered when designing a digester. Operating parameters such as HRT, loading rate, temperature regime, and TS content can all present themselves as important parameters in the design of digesters. Decreased digestion times should, in theory, be able to increase the volume of waste treated over a given time period in batch digesters, thus allowing greater biogas yields. However, the temperature may affect the time needed for digestion, with one study finding that 95% of the theoretical methane yield was attained in 11 days in a thermophilic batch digester as opposed to 27 days in a mesophilic batch digester [94]. However, these results should not be treated as a definitive guide for choosing a temperature regime; the ideal temperature regime for a batch digester can depend on a variety of other operational concerns. The TS content of a digester also presents itself as a parameter that merits consideration. While some digesters report that an increased TS content is able to increase biogas yield, digesters may be constrained by an upper TS content limit, above which biogas production may become compromised [95]. Our research group will synthesize the findings in these areas and determine the most practical operating parameters for our digester.

Finally, there exist numerous techniques of pretreating waste before digestion; the use of pretreatments can reduce the time needed in a digester. Fungal, chemical, mechanical, and thermal techniques have all been researched and are applied to different extents in practice; these pretreatments all share the underlying premise that digestion can be improved by increasing the solubility and particle size reduction [96]. While each is able to provide modest increases in biogas production, the investment and operating costs necessary for the pretreatment infrastructure must be properly evaluated before implementation [97]. The pretreatment of waste is one of many means by which the anaerobic digestion process may be expedited, and pretreatment may reduce the retention time needed for complete degradation of wastes in a batch reactor if the increased costs associated are justifiable [96]. However, due to the attractive lower costs of mechanical pretreatment, our research group will proceed with this form of pretreatment in our design of small-scale digester.

## 7. Conclusions

The rise of anaerobic digestion research has been facilitated by a drive for environmentally sustainable methods for waste management, and anaerobic digestion has already demonstrated its promise as a technology with a diverse range of applications from food waste, agriculture to wastewater treatment. While a great deal of understanding of the underlying science has been accumulated and many innovations in optimization proposed, there still exist gaps that impede researchers from having a complete understanding of the intricate process that underlies anaerobic digestion, especially for batch type digesters. In addition, the diverse applications of anaerobic digestion signify a continuing potential for research in optimization and increasing efficiency, in addition to reducing the time and costs associated. Factors pertaining to the design, pretreatment methods, and digester conditions of a small-scale batch digester should all be considered to ensure that an efficient and cost-effective final product is viable.
